# Supplementary material for: Content-rich biological network constructed by mining PubMed abstracts
Source: BMC Bioinformatics. 2004 Oct 8;5:147. doi: 10.1186/1471-2105-5-147 (PMC528731; doi:10.1186/1471-2105-5-147)
Supplement: Additional File 5 — The original Chilibot query results of the term "long-term potentiation (LTP)" and 22 other terms, limiting the latest references analyzed to the years 1990, 1995, 2000, and 2004. [file 1471-2105-5-147-S5.bz2 › chilibotAdditionalFile5/ltp2004/html/ATF.html]

 


**ATF** (Input: ATF ) 

---


**Google Searches:** Entire Web  | EDU domain only  | PDF files only

.

**External Links:** OMIM |
LocusLink
| Swissprot
| GeneCards

  
**Maps of ATF**

Simple
Complete
graph in
radiant
tree
square
layout.

**New Hypothesis !**

**Synonyms** 

- atf   [PubMed]

**Synopsis**

- These results suggest **ATF** 2 involvement in cell survival of dentate gyrus granule cells.  Acta Neuropathol , 2002 
  [23]

- Thus, DADLE stimulated phosphorylation of 71kDa **ATF** 2 and its association with JNK, suggesting that JNK is activated through DORs.  Cell Immunol, 2003 
  [19]

- ERK1 was demonstrated to be identical to a p46 c Jun **ATF** 2 kinase previously shown to be activated by reperfusion after a 60 min occlusion.  Brain Res Mol Brain Res, 2003 
  [18]

- Pretreatment of K562 cells with the JNK inhibitor, dicoumarol, abolished PBOX 6 induced phosphorylation of c Jun and **ATF** 2 and inhibited the induced apoptosis, suggesting that JNK activation is an essential component of the apoptotic pathway induced by PBOX 6.  J Biol Chem, 2002 
  [16]

- ATF3Activating transcription factor 3, a member of the CREB **ATF** family, can be induced by stress and growth factors in mammalian cells, and is thought to play an important role in the cardiovascular system.  Genes CellsGenes Cells, 2002 
  [16]

- Herein, we show that cadmium induces reactive oxygen species ROS that activate c Jun N terminal kinase JNK and p38 mitogen activated protein kinase MAPK and their substrates, activating transcription factor 2 **ATF** 2, CRE binding protein CREB and c Jun.  Cell Signal, 2004 
  [16]

- Both basal and **ATF** mediated migratory responses were suppressed in the presence of selective pharmacological inhibitors LY294002, U73122, and U0126, implicating the respective roles of phosphatidinylinositol 3 kinase PI 3 K, phospholipase C PLC, and MEK1 2 in basal and **ATF** stimulated migratory capacity.  Exp Cell Res< MedlineTA>Exp Cell Res, 2004 
  [15]

- Exposure to forskolin, which resulted in CREB and **ATF** 1 phosphorylation, caused a reversible decrease in cell volume 14.5 5%.  Exp Eye Res, 2004 
  [14]

- Taken together, these findings suggest that HTLV 1 Tax activates p21 Waf1 Cip1, a cell growth inhibitor, in a p53 independent mechanism through CREB **ATF** related transcription factors, and inhibits cdk2.  Int J Cancer, 2003 
  [13]

- Cross linking of CD158j resulted in selective phosphorylation of c Jun NH 2 terminal protein kinase JNK and its upstream kinase, MKK4 that led to the expression of **ATF** 2 and c Jun, all in the absence of extracellular signal regulated kinase ERK 1 2 phosphorylation.  J Exp Med, 2003 
  [11]

- Phosphorylated c Jun, JunB, JunD, and Fra 1,   FASEB J, 2003 
  [10]

- It comprises the X1, X2 and Y boxes, bound by RFX, X2 BP **ATF** CREB and NFY factors, respectively.  Immunology, 2004 
  [10]

- Regulationof Proglucagon Transcription by Activated Transcription Factor **ATF** 3 and a Novel Isoform, ATF3b, through the cAMP response Element **ATF** Site of the Proglucagon Gene Promoter.  J Biol Chem, 2003 
  [10]

- This chromatin structure alteration required components for the stress activated protein kinase SAPK cascade and both subunits of the M26 binding CREB **ATF** type protein Atf1.Pcr1.  Nucleic Acids Res, 2004 
  [10]

- CEP increased the expression and phosphorylation levels of c Jun and **ATF** 2 transcription factors.  Cell Signal, 2002 
  [10]
